# Semantic Grounding of Novel Spoken Words in the Primary Visual Cortex

**DOI:** 10.3389/fnhum.2021.581847

**Published:** 2021-02-24

**Authors:** Max Garagnani, Evgeniya Kirilina, Friedemann Pulvermüller

**Affiliations:** ^1^Department of Computing, Goldsmiths, University of London, London, United Kingdom; ^2^Brain Language Laboratory, Department of Philosophy and Humanities, WE4, Freie Universität Berlin, Berlin, Germany; ^3^Neurocomputational Neuroimaging Unit, Freie Universität Berlin, Berlin, Germany; ^4^Department of Neurophysics, Max-Plank Institute for Cognitive and Brain Sciences, Leipzig, Germany; ^5^Berlin School of Mind and Brain, Humboldt Universität Zu Berlin, Berlin, Germany; ^6^Einstein Center for Neurosciences Berlin, Berlin, Germany; ^7^Cluster of Excellence “Matters of Activity”, Humboldt Universität zu Berlin, Berlin, Germany

**Keywords:** embodied cognition, word learning, language acquisition, action-perception circuit, conceptual category

## Abstract

Embodied theories of grounded semantics postulate that, when word meaning is first acquired, a link is established between symbol (word form) and corresponding semantic information present in modality-specific—including primary—sensorimotor cortices of the brain. Direct experimental evidence documenting the emergence of such a link (i.e., showing that presentation of a previously unknown, meaningless word sound induces, after learning, category-specific reactivation of relevant primary sensory or motor brain areas), however, is still missing. Here, we present new neuroimaging results that provide such evidence. We taught participants aspects of the referential meaning of previously unknown, senseless novel spoken words (such as “Shruba” or “Flipe”) by associating them with either a familiar action or a familiar object. After training, we used functional magnetic resonance imaging to analyze the participants’ brain responses to the new speech items. We found that hearing the newly learnt object-related word sounds selectively triggered activity in the primary visual cortex, as well as secondary and higher visual areas.These results for the first time directly document the formation of a link between the novel, previously meaningless spoken items and corresponding semantic information in primary sensory areas in a category-specific manner, providing experimental support for perceptual accounts of word-meaning acquisition in the brain.

## Introduction

When a language is learnt, at least some of its novel symbols must be “grounded” in perceptions and actions; if not, the language learner might not know what linguistic symbols relate to in the physical world, i.e., what they are used to speak about, and, thus (in one sense) what they “mean” (Freud, 1891; Locke, 1909/1847; Searle, 1980; Harnad, 1990, 2012; Cangelosi et al., 2000). Indeed, children typically acquire the meaning of some words used to refer to familiar objects (such as “sun”) in situations involving the simultaneous perception of the spoken lexical item and the referent object (Bloom, 2000; Vouloumanos and Werker, 2009); similarly, it has been argued that a common situation for learning action-related words (like “run”) involves usage and perception of the novel items just before, after or during the execution of the corresponding movement (Tomasello and Kruger, 1992). Embodied theories of grounded semantics (Barsalou, 2008; Glenberg and Gallese, 2012; Pulvermüller, 2013) have long postulated that repeated co-occurrence of symbol and referent object (and/or action execution) leads to the emergence of associative links in the cortex, “cell assembly” circuits (Hebb, 1949) binding symbols (word-form representations emerging in perisylvian areas) with corresponding semantic information coming from the senses and the motor system (Pulvermüller and Preissl, 1991; Pulvermüller, 1999). This neurobiological version of semantic grounding makes one important prediction: as a result of learning, a link must be made between a word and corresponding sensory or motor brain patterns, so that the latter are—at least in some cases—reactivated upon word presentation. So, do specific aspects of the meaning of words become manifest in primary sensory and motor areas?

A body of neuroimaging results seems to demonstrate category related reactivation of sensorimotor cortices during word and sentence processing and comprehension (e.g., for reviews see Pulvermüller and Fadiga, 2010; Kiefer and Pulvermüller, 2012; Meteyard et al., 2012), thus providing some support for the existence of such functional links in the brain both in adults as well as in pre-school children (James and Maouene, 2009; Engelen et al., 2011; see Wellsby and Pexman, 2014 for a review). The majority of the studies in this area, however, used natural language stimuli (e.g., Binder et al., 2005); as it is very difficult to identify lists of words that are matched on all relevant psycholinguistic variables (Bowers et al., 2005) and individual circumstances are likely to play an important role in word learning processes (Kimppa et al., 2016), the presence of possible confounding factors cannot be entirely ruled out. For example, when just choosing words typically used to speak about tools or animals, any brain activation differences between these may be explained by the physical differences between the word stimuli chosen—which may be longer or shorter—or the psycholinguistic factor of word frequency (words from one category may be more common than those of the other). Although these factors could be controlled for, other factors, such as the frequency with which the words’ letters, phonemes or letter/phoneme-bigrams or -trigrams occur, the number of similar words (lexical neighbors), the size of their morphological family, their lexical category and fine grained grammatical features and countless other linguistic properties may also have an effect. Even worse: at the semantic level, the level of concreteness, imageability, relatedness to specific sensory and motor modalities may influence the brain response. In short, it is simply impossible to match for all relevant psycholinguistic features when considering utterances from natural languages, and, therefore, any studies on real words suffer from this “confounded nuisance” problem (Cutler, 1981).

One way to address this issue is to deploy novel, carefully designed speech stimuli in rigorously controlled learning experiments. This approach has been adopted in several behavioral (e.g., McKague et al., 2001; Smith, 2005; Leach and Samuel, 2007; Merkx et al., 2011; Brown et al., 2012; Szmalec et al., 2012; Tamminen et al., 2012; Henderson et al., 2013; Bakker et al., 2014; Hawkins and Rastle, 2016; Öttl et al., 2017) and neuroimaging studies (e.g., Clark and Wagner, 2003; Gaskell and Dumay, 2003; McLaughlin et al., 2004; Breitenstein et al., 2005; Dumay and Gaskell, 2007; Davis et al., 2009; Davis and Gaskell, 2009; Paulesu et al., 2009; Shtyrov et al., 2010; Shtyrov, 2011; Pulvermüller et al., 2012; Takashima et al., 2014; Bakker et al., 2015; Hawkins et al., 2015; Leminen et al., 2016) to investigate the mechanisms underlying word learning. Behavioral results (usually from a lexical decision or recognition tasks) have typically indicated the presence of competition effects between newly learnt items and previously existing words, taken as a hallmark of successful lexical competition and thus integration of the new item into the lexicon. Neuroimaging data obtained with different methods (fMRI, EEG, MEG etc.) generally revealed changes in brain responses to the trained items compared to untrained ones, the former becoming more “similar” to those induced by familiar words. Recent neurophysiological evidence also suggests that cortical memory circuits for novel words can emerge rapidly in the cortex (i.e., without a period of overnight consolidation; Shtyrov et al., 2010; Shtyrov, 2011; Yue et al., 2013), and even in absence of focussed attention (Kimppa et al., 2015).

**Figure 1:**
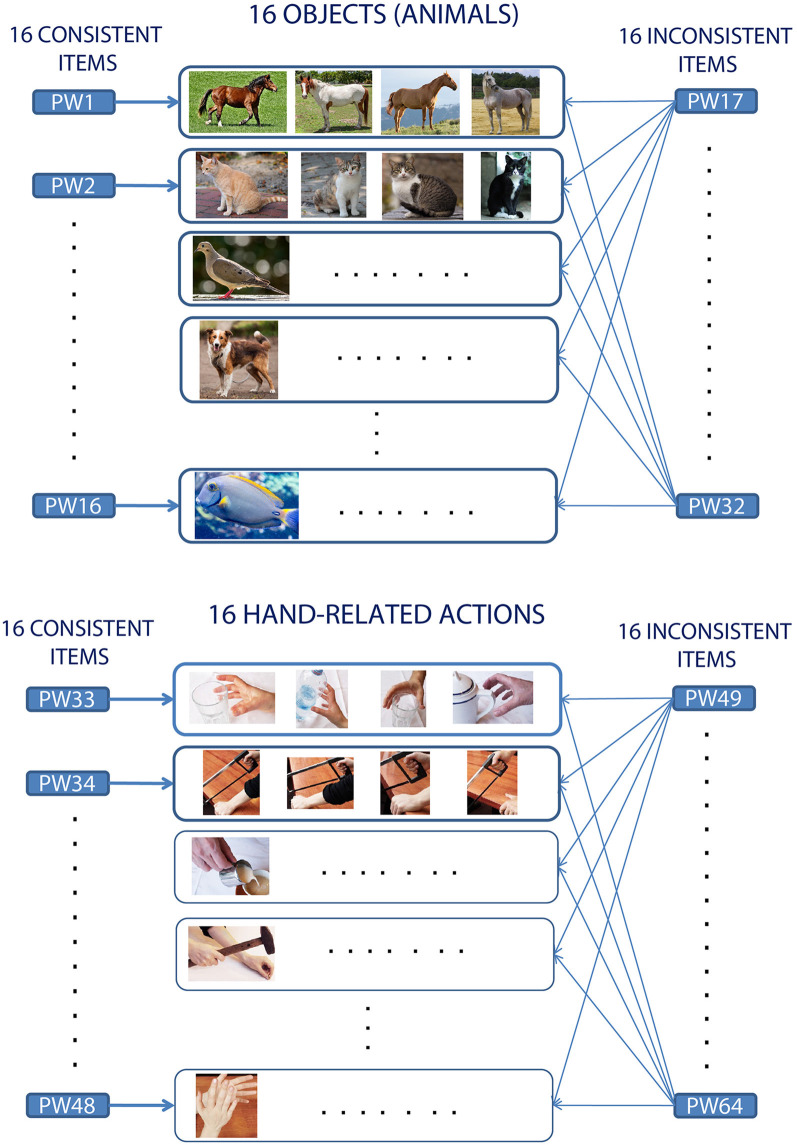
Experimental design and word-picture pairing in consistent and inconsistent learning conditions. The schema illustrates the generic mapping between the to-be-learnt spoken pseudowords (represented by the rectangles labeled PW1–PW64) and condition (Consistent vs. Inconsistent), and, accordingly, the correspondence (indicated by the arrows) between an auditory stimulus and the set of picture instances (rectangles in the middle) used to convey referential aspects of its meaning during the training. Note the resulting “1:many” mapping between word form and objects (or actions) from the same referent conceptual category (see main text for details).

Despite the abundance of studies documenting the emergence of neural correlates of novel spoken lexical items, only a few directly investigated the cortical mechanisms underlying the formation of a semantic link between a new word form and information about its meaning, manifest as neural activity in the brain’s perception and action systems. Several researchers successfully used associative learning to demonstrate that patterns of activity induced in the cortex by the perception of sensory items can be memorized and later reinstated in relevant modality-specific brain areas (including primary ones) using cued or free recall, in a category-specific manner (e.g., Breitenstein et al., 2005; Polyn et al., 2005; Kiefer et al., 2007; Mitchell et al., 2008; Kuhl and Chun, 2014; Vetter et al., 2014; Hindy et al., 2016; Horoufchin et al., 2018). However, none of these investigated the learning of *novel* (spoken or written) linguistic items, hence suffering from the confounded nuisance problem mentioned earlier. Moreover, crucially, in these studies subjects were typically trained to associate *one* *specific* cue stimulus with *one* (normally visual) stimulus, in a 1:1 (1-to-1) manner. Instead, when learning the meaning of a new word or symbol, the novel item usually co-occurs with several *instances* of the same concept it refers to. For example, a typical learning situation for a concrete word like “cat” will involve its repeated usage in concomitance with the visual perception of different exemplars of cats, having different size, color, etc. More abstract words (like “beauty”) might co-occur with objects from very different conceptual categories (e.g., human faces, flowers, statues, etc.; Pulvermüller, 2013). Therefore, in the real world the mapping between word forms and referent objects (or actions) is not 1:1, but, rather, “1*:many*.” The present study attempts specifically to reproduce this situation (see Figure 1). Hence, it improves upon the above-mentioned efforts in that it adopts: (1) carefully matched and previously meaningless, novel spoken items; and (2) a “1:many” mapping between a verbal label and associated (visual or motor) referent items.

Perhaps most relevant in the present context is the pioneering work by Breitenstein et al. (2005), in which increased left hippocampal, fusiform and inferior-parietal activity was observed in response to novel spoken items after these had been associated (1:1) with visual object pictures. Although this study did report the involvement of left inferior-temporal (fusiform gyrus) visual areas, no earlier (let alone primary) visual cortex activity was found. More recently, Liuzzi et al. (2010) successfully influenced the learning of novel body-related action words (again using a word-picture association task) by application of transcranial direct current stimulation (tDCS) to left motor cortex (MC) but not dorsolateral prefrontal cortex (DLPFC), thus providing evidence for the involvement of the former (and not the latter) areas in the word acquisition process. Furthermore, in an electroencephalography (EEG) study (Fargier et al., 2012), participants were repeatedly exposed to videos of object-oriented hand and arm movements (which they were asked to first watch and then mimic) and novel spoken word stimuli (presented during self-performed action). As a result of training, the authors found an increase in the motor-related brain activity (measured as the level of synchronization in the μ frequency band) over centro-parietal regions for the verbal stimuli (as well as for the videos), interpreted as indexing novel associations between newly learnt phonological representations and corresponding action-execution events (Fargier et al., 2012). The lack of an analysis of the underlying cortical sources, however, prevents this study from providing evidence of semantic grounding in the primary motor or somatosensory cortices.

In summary, while the above results, taken together, strongly suggest the involvement of sensorimotor areas in the acquisition of the meaning of new object- and action-related words, to date no learning study has been able to document the emergence of a link between a *novel* spoken item and associated semantic information in primary (visual or motor) brain areas.

Using event-related functional magnetic resonance imaging (fMRI) we aimed here at providing such evidence. We taught participants aspects of the referential meaning of 64 spoken pseudoword items, focusing specifically on the acquisition of novel object- and action-related words. Training—which took place over three consecutive days—involved repeated co-occurrence of the novel word sounds with either a familiar hand/arm-related action or a familiar object (animal) picture, using a 1:many mapping (see Figure 1). Word-picture matching and lexical-familiarity decision (FD) tests were used as behavioral measures of successful learning (for details see “Materials and Methods” section).

We hypothesized that, during word acquisition, Hebbian learning mechanisms induce the emergence in the cortex of lexicosemantic circuits linking phonological representations in frontotemporal perisylvian language areas with information coming from the visual or motor systems (Pulvermüller and Preissl, 1991; Pulvermüller, 1999). The category-specific distributions of such cell-assembly circuits (see Garagnani and Pulvermüller, 2016; Tomasello et al., 2017, 2018 for recent neurocomputational accounts) lead to the prediction that recognition of the newly-grounded language items should induce double-dissociated patterns of hemodynamic responses in the brain. More precisely, we predicted that auditory presentation of successfully learnt action-related words should selectively reactivate areas preferentially responding to the observation of arm/hand motion execution [including primary motor, premotor and higher areas in the frontoparietal system for action observation and recognition (Jeannerod, 1994; Fadiga et al., 1995; Gallese et al., 1996; Rizzolatti et al., 2001)], while object-related words should selectively trigger activity in areas involved in processing information related to visual-object identity [here, we expected primary and higher visual cortices in the occipito-temporal regions of the ventral visual stream (Ungerleider and Mishkin, 1982; Ungerleider and Haxby, 1994; Perani et al., 1995)]. To estimate what the former and latter areas corresponded to in the present study, we used a Visual Localizer task, during which all action- and object-related pictures were presented (for details see “Materials and Methods”, “fMRI Session-Procedures and Design” sections).

## Materials and Methods

### Subjects

Twenty-four healthy right-handed (Oldfield, 1971) monolingual native speakers of German (15 female) subjects aged between 18 and 35 participated in all parts of the experiment. They had no record of neurological or psychiatric diseases, vision or hearing problems and reported no history of drug abuse. All subjects gave their written informed consent to participate in the experiment and were paid for their participation. The experiment was performed following the Helsinki Declaration. Ethics approval had been issued by the ethics committee of the Charité University Hospital, Campus Benjamin Franklin, Berlin, Germany.

### Design

The to-be-learnt items consisted of 64 bi-syllabic phonotactically-legal meaningless word-forms (see Supplementary Material for a full list and physical features of the linguistic stimuli). Another 64 strictly matched pseudowords, not presented to the participants during the training and henceforth referred to as the “untrained” stimuli, were used as a baseline for the fMRI data analysis (see “Statistical Analysis” section for details) and as a control condition in the post-training behavioral testing [see “Lexical Familiarity Decision (FD) Test” section]. Using a fully orthogonal design, the experiment manipulated three factors: Consistency (“Consistent” vs. “Inconsistent”), WordType (“Action” vs. “Object”), and Training (“Trained” vs. “Untrained”). In the “Consistent” condition the pseudoword-to-referent-concept mapping was *1:1*—i.e., each pseudoword was associated with one particular basic conceptual category of objects or actions (see Figure 1). In the Inconsistent one, the mapping was *1:many* (i.e., each pseudoword was associated with 16 different familiar actions or 16 different objects). Thus, the referential meaning of a Consistent pseudoword was similar to a basic category term (such as “dog” or “grasping”), whereas Inconsistent pseudowords were used similarly to a general category term (such as “animal” or “performing an action”). Note that the same object (or action) referent co-occurred with 17 different novel linguistic forms (one Consistent and 16 Inconsistent ones); also, each novel word was paired either with four instances of the same basic concept (e.g., four exemplars of a dog, or four instances of grasping) or with many different objects or actions (16 animals or 16 hand actions). This effectively results in a “1:many” mapping between word forms and referent items. Details about the familiar objects and hand actions chosen, and representative examples of corresponding visual stimuli, are provided in Supplementary Material.

### Procedures

The experiment unfolded over four consecutive days (DAY1–DAY4): participants underwent training during DAY1–3 and fMRI scanning on DAY4. The training was delivered in three sets of two sessions, each session lasting about 1 h and consisting of four blocks of 256 randomly ordered trials. In each (3.6-s long) trial one of the spoken words to be learnt was presented together with a picture of the corresponding referent object or action. An inter-stimulus interval (ISI) of 2.75 s followed, during which a blank screen was shown. Each of the 64 words was presented 16 times per session; more precisely, each consistent word was paired four times with each of the four pictures of possible basic-category term referents (e.g., four dogs of different breeds), while each inconsistent word was paired (once) with all 16 items forming the “larger,” superordinate semantic category (i.e., animals; see Figure 1). We ensured that each of the 128 pictures (four instances of 16 object and 16 action types) occurred exactly eight times/session, appearing four times in a consistent- and four times in an inconsistent-word context. Participants were instructed to pay full attention to both sounds and images and were allowed to pause before the start of each new block (lasting approximately 15′22”) and to take a 5–10 min break between two consecutive sessions. Thus, each word and picture was presented the same number of times (16 for words, eight for pictures) and only the word-picture pairing scheme differed between conditions.

At the end of each day of training, as well as after scanning, subjects were administered a Word-to-Picture matching (WTPM) test, aimed at assessing their ability to acquire and retain the referential meaning of the novel words throughout the experiment. On DAY4, after the scanning session, all participants underwent a lexical familiarity decision (FD) test, followed, once again, by a WTPM test (see below for details).

During all parts of training and behavioral testing, subjects were wearing headphones and were seated in front of a computer screen in a quiet environment. Stimulus delivery was controlled by a personal computer running E-prime software (Psychology Software Tools, Inc., Pittsburgh, PA, USA); auditory stimuli were delivered binaurally at a comfortable hearing level through professional headphones. In the scanner, speech stimuli were delivered using the fMRI-compatible sound-stimulation system VisuaStimDigital (Resonance Technology Inc., Northridge, CA, USA) and auditory and visual delivery was controlled by a personal computer running Presentation software (Neurobehavioral Systems, Inc., Berkeley, CA, USA).

#### Word-to-Picture Matching (WTPM) Test

Each of the 64 trials started with a fixation cross displayed in the center of the screen for 900 ms and simultaneous auditory presentation of one of the 840 (ms long) spoken words participants had been learning. After 900 ms, the fixation cross was replaced by two pictures (positioned on the left- and right-hand sides of the screen), depicting the correct referent (object or action) for that word and a distractor item or “lure”. The lure was randomly chosen from the same semantic category as the target if this was a “consistent” item, and from the “incorrect” superordinate category otherwise (i.e., an object for an action-word target and an action for an object-word one). Subjects were instructed to indicate which picture—the one on the left or right—matched the correct meaning of the word by pressing one of two buttons using their left-hand middle (indicating “left”) or index fingers (indicating “right”); they were asked to be as quick and accurate as possible. The two images were displayed for up to 3.6 s and the subjects’ first response and reaction times (RT) were recorded. Target position was randomized. After each button press, participants were provided with immediate feedback about the correctness of their choice in the form of an iconised face (shown during the ISI, 500 ms long), indicating a correct (“smiling” face) or an incorrect (“frowning” face) response. In case no response was given during picture display, the “frowning” face appeared. A final overall score (% of correct and no-response trials) was displayed on the screen at the end of the test (which lasted up to 5′ 20” in total).

#### Lexical Familiarity Decision (FD) Test

In this test, participants heard the trained 64 pseudowords randomly mixed with other 64 closely matched, untrained items (see Supplementary Material), and had to judge whether the stimulus presented was one of those they had been learning (“old”) or not (“new,” or “untrained”). The “old” items had been heard 96 times during the preceding 3 days, and four additional times in the scanner. The “new” ones had been heard only four times in the scanner (control). The speeded task thus involved 128 randomly ordered trials. Each trial started with a fixation cross, 500 ms upon which a spoken word was played. Nine hundred millisecond after each spoken word onset, the fixation cross disappeared and participants were given up to 3.6 s to decide whether the stimulus they had heard was one of the learnt, “familiar” ones or not and hence make either a left- or a right-button press. Assignment of buttons to response types was counterbalanced across subjects. Accuracies and reaction times were collected. This procedure contained 128 trials with stimulus onset asynchronicity (SOA) ≤5.0 s and thus a maximal test duration of 10′ 40”.

#### Analysis of the Behavioral Data

For the word-picture matching test, we computed hit and false-alarm (FA) rates for each participant on each of the repeated tests (administered once on each training day and once after scanning), as well as hit RTs; to exclude any effect of response bias on the results hit and FA rates were then used to calculate the sensitivity index, or d’ (Peterson et al., 1954). As we expected participants’ performance to improve with training and to be generally higher for novel Consistent words than Inconsistent ones, we tested for the presence of training and consistency effects (and their possible interactions) by subjecting d’ and RTs data to repeated-measure analyses of variance (ANOVAs) with factors TestingDay (DAY1, DAY2, DAY3) and Consistency (Consistent, Inconsistent).

Similarly to the above analysis, for the lexical-decision test, we also computed each participant’s hit and FA rates, as well as hits and correct-rejections RTs. To test for possible effects of the semantic category (i.e., WordType) and consistency on the ability to recognize the newly learnt words, d’ values were then calculated under four different conditions: Consistent-Action, Consistent-Object, Inconsistent-Action and Inconsistent-Object items; to compute these values, we used the same FA rates obtained from the analysis of the responses to the 64 untrained items (all equally “unknown” and not subject to further subdivisions). Both sets of data were then subjected to repeated-measure ANOVAs with factors WordType (Object, Action) and Consistency (Consistent, Inconsistent). The statistical analyses were performed using Statistica v.12 software (StatSoft, Tulsa, OK, USA) and results were Greenhouse–Geisser corrected for non-sphericity where appropriate.

### fMRI Session

#### Procedures and Design

In the scanner, subjects underwent four runs (Runs 1–4) of auditory stimulation, followed by one Visual Localizer run (with no auditory stimuli). They were instructed to fixate a cross on the screen center and pay full attention the speech sounds during auditory stimulation, and to focus their attention on the visual display during the Visual Localizer run. Throughout the scanning, we ensured that participants were awake by monitoring their eyes *via* MR-compatible camera (EyeLink 1,000 Plus, SR-Research TDD., Mississauga, Canada). An event-related design was used for auditory Runs 1–4; each run contained 128 events involving the auditory presentation of one of the 128 spoken stimuli (64 trained plus 64 untrained), mixed with 32 “null” (or silent) events. Each event was 840 ms long and was followed by an inter-stimulus interval which varied randomly between 1.16 and 2.16 s (so that SOA varied randomly between 2.0 and 3.0 s). The order of the condition sequence was optimized in each of the four runs using the freely-available Optseq2 software^1^. As the assignment of stimulus sets to conditions was fully counterbalanced across subjects, we used the same four stimulus sequences for all subjects (counterbalancing run order). Each run lasted 7’ 12” and was followed by a short (approximately 2 min) break during which we checked that participants were doing fine and could hear the stimuli clearly. We also asked them whether they recognized a given item as one of those they had just heard in the last session (this one stimulus was chosen at random from the set of items just presented).

The Visual-localizer task adopted a blocked design and involved the visual presentation of all 128 pictures used during the training, plus their 128 “blurred” versions. Stimuli were delivered in four sets of four blocks in a Latin-square design, each set containing 16 objects, 16 actions, 16 blurred-object and 16 blurred-action pictures presented for 1 s each. Within-block order was randomized. Each set of 4 blocks was preceded by 16 s of the fixation-cross display, leading to a total duration of approximately 3′ 40”.

#### MR Acquisition and Preprocessing

fMRI measurements were performed on a 3 T TIM Trio (Siemens, Erlangen, Germany, Software VB17) MRI scanner, using a 12-channel radio-frequency (RF) receive head coil. The 2D echo-planar imaging (EPI) sequence with *T*_R_ / *T*_E_ = 2 s/ 30 ms, field of view (FOV) = 192 mm, matrix size = (64 × 64), in-plane resolution 3 × 3 mm^2^, fat saturation, a readout bandwidth (BW) = 2,232 Hz/Px and echo spacing (ES) = 0.53 ms. was used for fMRI recording. Thirty-seven 3 mm thick slices oriented along the anterior commissure (AC)—posterior commissure (PC) anatomical axis with an inter-slice gap of 20% were recorded in an interleaved order, using the anterior-posterior (A-P) axis as phase-encoding (PE) direction. Parallel imaging with an acceleration factor (AF) = 2 was used along the PE direction. Images were reconstructed using the generalized autocalibrating partially parallel acquisitions (GRAPPA) method (Griswold et al., 2002) using 24 reference lines. Field map was acquired using gradient-echo sequence with two echo times *T*_E1_/*T*_E2_ = 4.9 ms./7.4 ms. Anatomical images were acquired using *T*_1_–weighted anatomical images (MPRAGE *T*_R_/*T*_E_/*T*_I_/BW = 2,300 ms/3.03 ms/900 ms/130 Hz/Px, 1 × 1 × 1 mm^3^ resolution) at the end of the scanning session.

The fMRI data were analyzed using SPM8 software^2^. EPI images were first corrected for the different timing of the slice acquisition by temporal interpolation to the acquisition time of the slice in the center of the volume using the standard method in SPM8. The images were realigned and unwarped, using the Realign and Unwrap function of SPM8 and the recorded field maps. Images were then normalized to the Montreal Neurological Institute (MNI) template (Mazziotta et al., 2001). The MNI normalization was performed based on the anatomical *T*_1_-weighted image, which was co-registered to the mean time-series EPI image. Finally, normalized images from all EPI sequences were smoothed with a Gaussian kernel full width at half maximum of 8 mm.

#### Statistical Analysis

Pre-processed images of each subject and all four EPI sequences underwent a fixed-effects general linear model (GLM) analysis. The GLM included eight functional predictors (corresponding to three independent factors WordType, Training, Consistency) and six nuisance predictors including rigid-body motion parameters extracted by the motion correction algorithm. Functional predictors were simulated by convolution of the standard SPM hemodynamic response function with boxcar functions corresponding to the presentation time of the respective pseudowords.

Analyses on the data from auditory stimulation Runs 1–4 were performed for eight contrasts. The first contrast “Speech vs. Silence” included all functional predictors (all pseudowords, “trained” and “untrained”) contrasted to the baseline. The other seven contrasts tested all possible main effects and two- and three-way interactions of the factors Consistency, Training and WordType. Functional predictors for the Visual-localizer run were simulated by convolution of standard SPM hemodynamic response function with boxcar functions corresponding to the presentation time of the respective blocks of images. Four contrasts were analyzed: “Action pictures vs. Object pictures,” “Object pictures vs. Action pictures,” “(Action pictures—Blurred Action pictures) vs. (Object pictures—Blurred Object pictures),” and “(Object pictures—Blurred Object pictures) vs. (Action pictures—Blurred Action pictures).”

The contrast maps for each contrast and volunteer were entered in the second-level random-effects analysis. The following random-effects group analysis estimated *t*-maps for the group from the previous single-subject contrasts. The *t*-maps were thresholded at the uncorrected voxel-wise significance level of *p* < 0.001. The correction for multiple comparisons was performed on the cluster level. Activation clusters were regarded as significant if they reached a peak- and cluster whole-brain family-wise error (FWE)-corrected level of *p* < 0.05.

#### Region-of-Interest Analysis

Our main hypothesis was that, across learning, mechanisms of Hebbian plasticity link patterns of neural activity related to word form processing with object and action information. Thus, activity in cortical regions strongly responding to hand-related pictures were expected to link up with the emerging phonological representations of the novel action words; likewise, areas preferentially responding to objects pictures should be recruited during the semantic grounding of the novel object-related words. Thus, as a result of word learning, we expected the brain responses to the newly acquired spoken items to exhibit double-dissociated patterns of activity in these areas. To test this hypothesis, we carried out a region of interest (ROI) analysis based on the data from the Visual-localizer task, as described below.

Two sets of ROIs were defined in MNI space as clusters of significant activation obtained in the second-level analysis from the two visual-localizer contrasts “Action pictures > Object pictures” (A) and “Object pictures > Action pictures” (B). These (disjoint) sets of areas exhibited preferential activation to either action or object, pictures, respectively. More precisely, from the contrast (B), two activation clusters in left and right primary visual cortex (labeled “d” in Figure 6) were used to define two ROIs which were selective for object pictures. From the other contrast (A), six ROIs were identified, based on two clusters emerging in the parietal cortex (labeled “c” in Figure 6) and two larger clusters spanning over multiple areas in occipital and posterior temporal cortices (“a” and “b”). As clusters “a” and “b” actually constituted a single cluster in the left hemisphere, but not on the right, the corresponding two ROIs [labeled “Left MOG” and “Left EBA,” MOG = middle occipital gyrus, EBA = extrastriate body area (Downing et al., 2001)] were defined by a cross-section of the larger activation clusters with spheres centered at the two sub-clusters’ local maxima. The same approach was used to define the two ROIs for clusters “a” and “c” on the right (labeled “Right MOG” and “Right Parietal + PCG”, PCG = precentral gyrus), which also merged into a single cluster. Spheres’ diameters (varying between 17 and 25 mm) were chosen to maximize the number of voxels from the relevant sub-clusters that would be included in the ROIs while keeping all sphere volumes disjoint. Brain responses to trained items were extracted from all eight ROIs. To statistically test for possible differences in ROI activation between semantic categories, data from four of these regions—two in each hemisphere, labeled “(Left / Right) V1/FFG” (FFG = fusiform gyrus) and “(Left / Right) EBA”—were submitted to a single ANOVA analysis with factors Hemisphere, WordType, Consistency and ROI. The choice of these two pairs of ROIs was based on our initial hypothesis, i.e., that areas preferentially responding to hand-related action pictures and areas selective to pictures of visual objects should show double-dissociated brain responses to auditory presentation of newly learnt action- or object-related spoken words. Again, all the statistical analyses were performed using the Statistica v.12 software (StatSoft, Tulsa, OK, USA).

**Figure 2:**
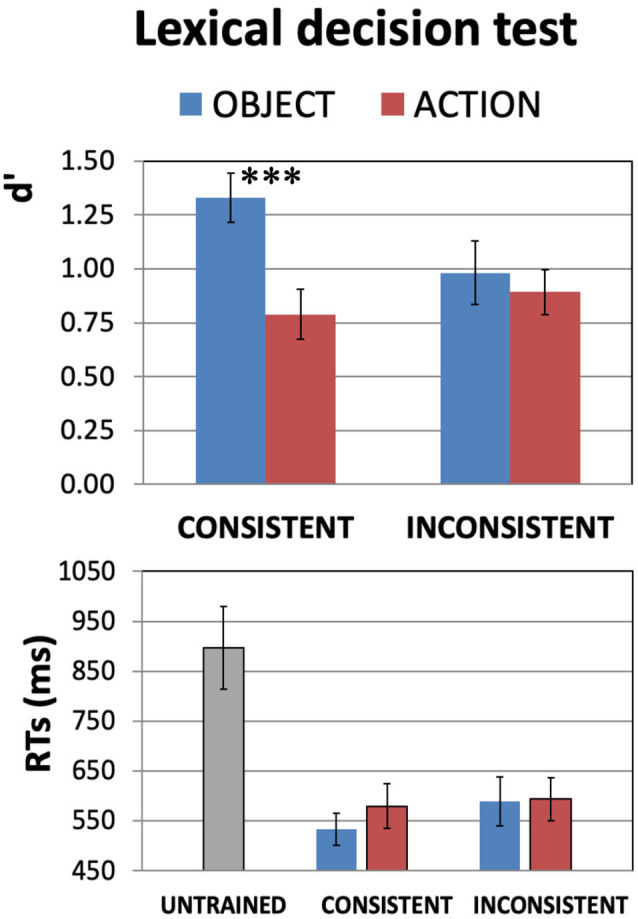
Results of the (auditory) word recognition test for the newly learnt words after training (**DAY4**). Experiment participants were asked to discriminate the 64 spoken items they had been learning from other 64 closely matched untrained pseudowords. Average *d*′ values **(Top)** and reaction times (RTs; **Bottom**) are plotted in the four different conditions. Recognition ability (Top plot) was generally above chance level (i.e., zero). Also note the significant Consistency-by-WordType interaction (*F*_(1,20)_ = 4.8, *p* = 0.04), seemingly driven by the better sensitivity to consistent object- than to consistent action-related words (confirmed by *post hoc* tests—see main text). As it is generally agreed that *d*′ values of 0.3 are to be considered “low,” 0.5 “medium,” and 0.8 and above “high,” even for action words a medium-to-high recognition performance was achieved. The generally shorter RTs (Bottom plot) for the correct detection of all trained items vs. rejection of untrained ones (*t*_(20)_ = 6.33, *p* < 0.000004) provide evidence that the training has induced the previously unknown speech items to acquire lexical status (error bars indicate standard errors, SE). ***p < 0.005.

**Figure 3:**
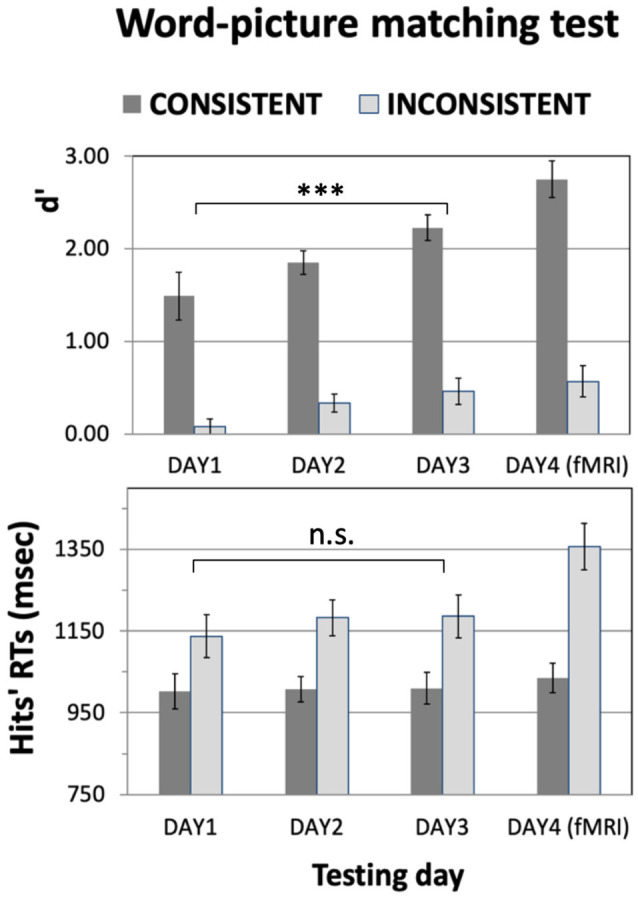
Results of the Word-to-Picture-Matching test as a function of training. Participants’ ability to identify the correct meaning of the newly learnt words was assessed using a two-alternative-forced-choice test administered at the end of each training day (**DAY1–DAY3**) and on the final day of the experiment (**DAY4**), after the fMRI scanning session (see main text). The to-be-learnt items included 32 consistent- and 32 inconsistent-meaning words, split equally into action- and object-related words. *D*′ values **(Top)** and hit RTs **(Bottom)** are plotted across the testing day. The protracted training produced a steady increase in performance (Top); there was no evidence of correspondingly slower RTs (Bottom), indicating that the better results were not a trivial effect of trading time for accuracy. Also note the better performance on items with a consistent than inconsistent meaning, which is in line with the chosen experimental design: unlike the consistent ones, inconsistent items were not associated to a single semantic category but to many different ones (see Figure 1 and main text); this made them significantly harder to learn. Error bars represent SE. ***p < 0.005. n.s., non significant.

Figure 3 plots the results they obtained on the word-picture matching test (averaged across 21 subjects). A 2 × 3 ANOVA with factors Consistency and TestingDay run on the d’ data from DAY1 to DAY3 reveals a main effect of TestingDay (*F*_(2,40)_ = 10.8, *p* = 0.0002) and of Consistency (*F*_(1,20)_ = 151.8, *p* < 0.1E-9), but no interaction between these factors (*F*_(2,40)_ = 0.78, *p* > 0.46, n.s). An analogous 2 × 3 ANOVA run on the RT data reveals a main effect of Consistency, with generally larger RTs for inconsistent than for consistent items (*F*_(1,20)_ = 82.6, *p* < 0.2E-7), but no effects of TestingDay (*F*_(2,40)_ = 0.18, *p* > 0.83, n.s.) or TestingDay-by-Consistency interactions (*F*_(1,20)_ = 0.60, *p* > 0.55, n.s). Planned comparisons on d’ data collapsing consistent and inconsistent conditions confirmed that performance generally improved throughout training, with d’ values larger on DAY2 than on DAY1 (*t*_(20)_ = 3.63, *p* = 0.002) and on DAY3 than on DAY1 (*t*_(20)_ = 5.18, *p* < 0.00005); overall performance did not significantly change between DAY3 and DAY4, the day of the fMRI scanning (*t*_(20)_ = 1.26, *p* > 0.22, n.s.).

**Figure 4:**
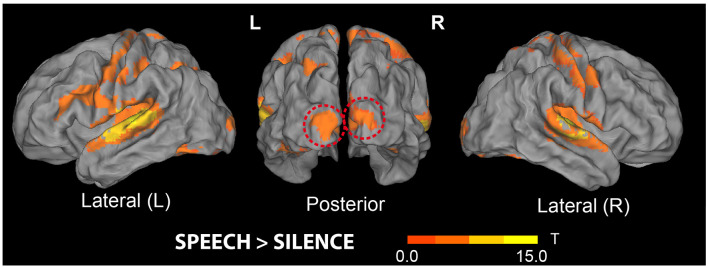
Brain areas showing increased responses to all (trained and untrained) pseudoword sounds compared with baseline. Stimuli included the novel 32 action- and 32 object-related words participants had been hearing over the preceding 3 days, mixed with 64 matched pseudowords never presented before (see “Materials and Methods” section). Note the significant clusters of activity increase in both left and right superior temporal gyri and the cluster emerging in bilateral primary visual cortex (middle, dashed red lines); the latter did not reach significance at the whole-brain level in this contrast—see also Table 1 (*t*-maps thresholded at uncorrected voxel-wise level *p* < 0.001, *T* = 3.58).

**Figure 5:**
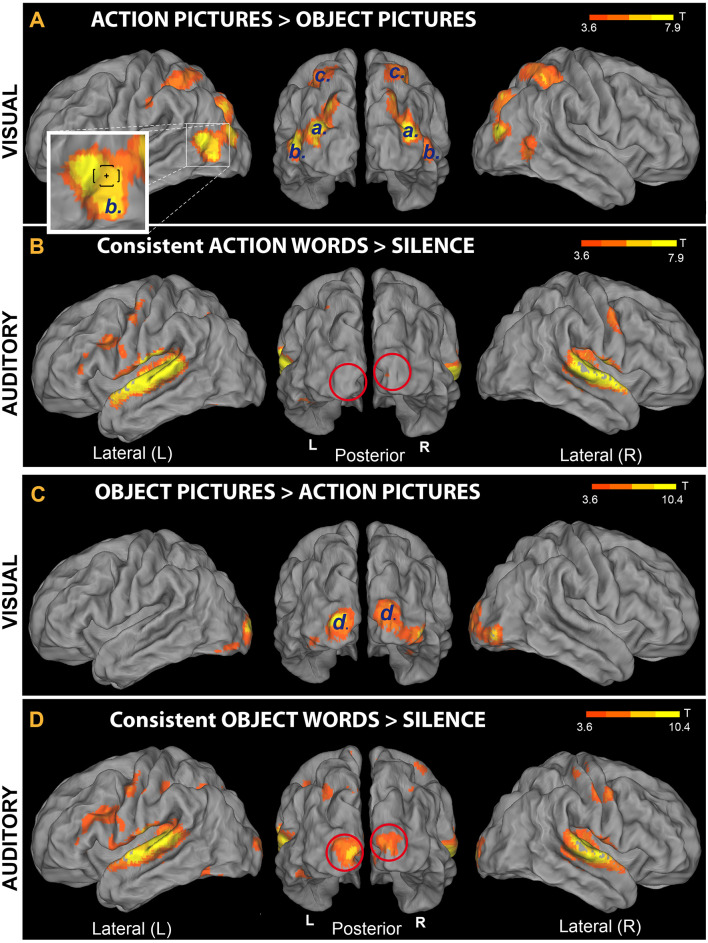
Comparison between brain responses to action and object pictures and responses to auditory presentation of newly learnt words. **(A,C)** Activation induced by familiar objects (animals) and familiar hand-related action pictures (data from the Visual-localizer task). The set of visual stimuli included all pictures that had been used to teach participants the novel words’ meanings (see “Materials and Methods” section). **(A)** Areas exhibiting preferential activation for action than object pictures; six clusters (labeled “[*a*]”, “*b*” and “*c*”) were identified. The lower-left inset shows an enlargement of the left hemisphere’s cluster “b”; note, within this cluster, the location of extrastriate body areas (EBA’s) main peak (Downing et al., 2001), indicated by a small cross and brackets [corresponding to average Montreal Neurological Institute (MNI) coordinates ± standard deviation, respectively]. **(C)** Areas showing increased sensitivity to object compared to action pictures; two clusters (labeled “*d*′’) were identified in left and right V1, extending to secondary and higher visual areas (BA 19, BA 37) bilaterally. **(B,D)** Presentation of the newly learnt words (data from Runs 1 to 4). Note that perception of novel word sounds having (consistent) object meaning sparked *primary visual cortex* bilaterally (panel **D**, red circles). This pattern reproduced activity increases specifically associated with the visual perception of corresponding object pictures (panel **C**). By contrast, consistent-action words **(B)** failed to reactivate V1, as predicted (all *t*-maps thresholded at voxel-wise level *p* < 0.001, uncorrected).

**Figure 6:**
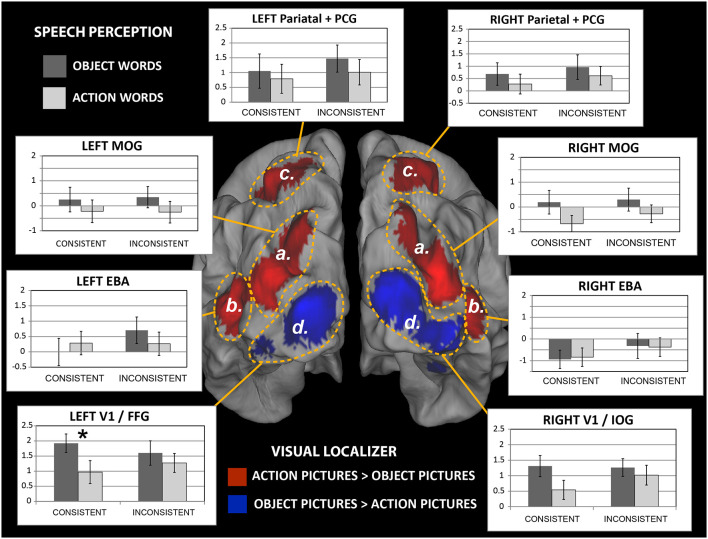
Brain responses to newly-learnt spoken words in the different regions of interest (ROIs). Middle: activation clusters resulting from analysis of the Visual-localizer data (see Figure 5, panels **A,C**) rendered onto a 3-D cortical surface (posterior view). Areas indicated by dashed yellow lines schematically identify ROIs boundaries. **Bar plots**: average % signal change induced by the auditory presentation of the novel spoken words that participants had been learning is plotted for each word category and ROI (error bars indicate SE). Note the significantly larger brain responses to consistent-object than consistent-action word sounds in the left hemisphere’s V1/FFG region, which includes parts of primary visual cortex and higher visual areas (fusiform gyrus). The same trend also emerged in the V1/IOG region on the right, although the difference there only approached significance (*F*_(1,19)_ = 4.3, *p* = 0.052, n.s.). Abbreviations as in Table 2. *p < 0.05.

## Results

To remove outliers from the lexical decision task data, we excluded any subjects whose average RTs were further than 2 SD from the group mean. This led to the identification of two participants (#2, #19). As the (hit) RTs alone cannot reveal whether participants have successfully learned the novel words, we also looked at d’ values (indexing the ability to discriminate trained from untrained items). All participants with a square-root transformed d’ value lower than 2 SD from the mean (#2 and #20) were also removed. In sum, subjects #2, #19 and #20 were excluded from any further analyses.

### Behavioral Results

Figure 2 reports the results of the lexical-decision test, administered on DAY4 after the scanning session, averaged across all subjects. The 2 × 2 ANOVA with factors WordType and Consistency run on the *d*′ data (top plot) revealed a significant WordType-by-Consistency interaction (*F*_(1,20)_ = 4.8, *p* = 0.04). There was also a main effect of WordType (*F*_(1,20)_ = 8.1, *p* = 0.010), with *d*′ values generally higher for the object- than for action-related items, but no main effect of Consistency (*F*_(1,20)_ = 1.96, *p* > 0.17, n.s). A similar 2 × 2 ANOVA run on the trained-only subset of the RTs data (bottom plot) revealed no significant effects of either WordType or Consistency (all *F’s*_(1,20)_ < 2.70, *p* > 0.11, n.s.).

Planned comparisons carried out on the *d*′ data of Figure 2 (top) indicate that, amongst the items with a consistent meaning, object-related words were recognized more easily than action-related ones (*t*_(20)_ = 3.57, *p* = 0.002), and that newly-learnt object words were better discriminated when they had a consistent meaning than an inconsistent one (*t*_(20)_ = 2.68, *p* = 0.014). *Post hoc* *t*-tests on the RT data revealed no significant differences in detection speed between consistent-object and consistent-action-related words (*t*_(20)_ = 1.35, *p* > 0.19, n.s.) or inconsistent-object ones (*t*_(20)_ = 1.70, *p* > 0.10, n.s.).

Overall, these results indicate that participants were not only able to recognize the newly learnt words (Figure 2) and discriminate them from similarly sounding, untrained ones (see Supplementary Material), but also to learn and generally retain the referential meaning of the novel speech items (Figure 3).

### Imaging Results

#### Whole-Brain Analysis: Runs 1–4

The results of the contrast “Speech > Silence” (see Figure 4) revealed significant clusters in the left and right superior temporal gyri, right cerebellum, and bilateral hippocampi (MNI coordinates for peak voxels showing increased activity are reported in Table 1 below). None of the 7 contrasts used for testing possible effects of the factors WordType, Consistency and Training produced a significant result, except for the main effect of Training and a main effect of Consistency. More precisely, the contrast “Trained > Untrained” revealed a cluster localized to the left middle occipital gyrus (MNI coordinates of the peak voxel: *x* = −40, *y* = −78, *z* = 32 mm, *T* = 6.86, *K_E_* = 1,256), which was marginally significant at peak-level (FWE-corrected, *p* > 0.053, n.s.). The “Inconsistent > Consistent” contrast produced a smaller (*K_E_* = 174) cluster localized to the right supramarginal gyrus (peak-voxel MNI Coord.: *x* = 62, *y* = −24, *z* = 26 mm, *T* = 4.78), not significant at peak-level (FWE-corrected, *p* > 0.071, n.s.).

**Table 1:** Results of Runs 1–4 (perception of spoken pseudowords).

Location	Peak voxel coordinates	*T*	Cluster size (voxels)
	(*x, y, z* mm)		
**Right HG**	**46, −20, 12**	**17.17**	**4,535**
Right STG	54, −22, 8	16.31	
Right HG	48, −12, 6	14.45	
**Left STG**	**−52, −24, 10**	**15.25**	**10,349**
Left STG	−64, −22, 8	14.74	
Left HG	−40, −26, 12	13.32	
**Right Cerebellum**	**26, −60, −28**	**9.28**	**7,702**
Right Cerebellum	34, −64, −28	
Right Cerebellum	6, −82, −34	9.13	
**Left Hippocampus**	**−10, −28, −10**	**7.67**	**204**
**Right Hippocampus**	**18, −30, −4**	**7.06**	**347**

*MNI coordinates for peak voxels showing increased activity for the contrast “Speech > silence” (significant both at cluster- and voxel-level, *p* < 0.05, FWE-corrected). Up to three peaks/cluster, more than 8.0 mm apart are reported (main peak in bold). HG, Heschl gyrus; STG, superior temporal gyrus*.

#### Whole-Brain Analysis: Visual Localizer

Analysis of the data from the Visual-localizer task (perception of object and action pictures) revealed several clusters of activity (Table 2). The “Action pictures > Object pictures” contrast produced three pairs of clusters bilaterally (labeled “a”, “b” and “c” in Table 2 and Figure 5A). Clusters “a” were localized to the (left and right) middle occipital gyri; clusters “b” emerged in the posterior parts of the middle temporal gyri, a region known as “extrastriate body area” (EBA; Downing et al., 2001); clusters “c” were localized to the parietal cortex and included a peak in the postcentral gyri (bilaterally). The reversed contrast (“Object pictures > Action pictures”) revealed two significant clusters, one—on the left—localized to the posterior segment of the middle occipital gyrus (primary visual cortex, BA 17) and extending to the fusiform gyrus (BA 19 and 37), and one—on the right—having a main peak located at the boundaries of the superior occipital gyrus and cuneus (BA 17) and a second—comparably strong—peak in the inferior occipital gyrus (BA 19).

**Table 2:** Results of the visual-localizer task.

Location	Peak voxel coordinates	*T*	Cluster size (voxels)
	(*x, y, z* mm)		
**(A) ACTION pictures > OBJECT pictures**
[*a*]. R MOG**	**30, −80, 12**	**9.5**	**3,123**
[*a*]. R MOG	30, −86, 34	6.4	
[*c*]. R Superior PL	22, −54, 58	6.3	
[*a*]. L MOG**	**−28, −86, 12**	**8.9**	**2,778**
[*b*]. L MTG (EBA)**	−50, −66, 8	7.8	
[*a*]. L MOG**	−22, −76, 32	6.6	
**[*b*]. R MTG (EBA)**	**48, −56, 6**	**6.1**	**585**
[*b*]. R ITG	52, −62, −2	5.8	
**[*c*]. L Inferior PL**	**−28, −48, 54**	**5.6**	**1,044**
[*c*]. L Superior PL	−30, −52, 60	5.6	
[*c*]. L PCG	−34, −36, 46	5.1	
**(B) OBJECT pictures > ACTION pictures**
[*d*]. L MOG (V1)**	**−18, −102, 6**	**10.6**	**977**
[*d*]. L FFG	−38, −72, −16	4.75	
[*d*]. RCuneus / SOG (V1)**	**18, −100, 16**	**8**	**1,069**
[*d*]. R IOG	46, −84, −6	7.9	

MNI coordinates for peak voxels showing increased activity for the “*Action > Object*” and “*Object > Action*” contrasts. Up to 3 peaks/cluster more than 8.0 mm apart are reported (main peak in bold). Activations are significant at cluster-level (*p* < 0.05 FWE-corrected); those marked **are also peak-level significant (*p* < 0.05 FWE-corrected). Letters in square brackets indicate corresponding activation clusters shown in Figure 5. R, right; L, left; IOG / MOG / SOG, inferior / middle / superior occipital gyrus; PCG, postcentral gyrus; ITG / MTG, inferior / middle temporal gyrus; PL, parietal lobule; FFG, fusiform gyrus; EBA, exstrastriatal body area; V1, primary visual cortex (BA 17).

Figure 5 shows cortical-surface renderings of the results obtained from the analysis of Visual-localizer data (panels A and C); results from two additional contrasts (*“Consistent Action words > Silence*” and* “Consistent Object words > Silence*”) performed on the data from Runs 1 to 4 are also reported there (panels B and D, respectively). This figure enables direct comparison of brain responses to auditory presentation of the spoken pseudowords participants had been learning over the preceding days with responses to the (action and object) pictures used during the training to convey aspects of the referential meaning of these novel items. In line with the results of the “Speech > Silence” contrast (Figure 4), both novel consistent-action and consistent-object words activated the superior temporal gyri bilaterally, as well as left and right hippocampi and cerebellum (not shown in the figure). However, the two semantic categories induced different responses in primary visual cortex (see red lines in panels B and D). In particular, object- (but not action-) related novel spoken words reactivated V1 bilaterally (MNI coordinates of the voxel showing the local maximum of activity for the V1 cluster were: *x* = −6, *y* = −102, *z* = 2 mm, *T* = 8.1), reproducing part of the response induced in V1 by visual perception of corresponding object pictures (see clusters “d” in panel C). None of the regions showing preferential responses to action pictures (panel A) appeared to be significantly reactivated by the perception of trained action-related items. The dissociation revealed by these contrasts was confirmed statistically by the results of the ROI analysis (see below).

#### Region-of-Interest Analysis

Brain responses to the trained items (consistent and inconsistent action- and object-related words) were extracted for each of the eight activation clusters defined based on the visual-localizer contrasts (labeled “a,” “b,” “c” and “d” in Table 2 and Figure 5). Preliminary inspection of the results revealed the presence of one outlier in the data set, exhibiting negative % signal change in all regions of interest; data for this participant (#11) were excluded from all subsequent statistical analyses, which was thus based on 20 subjects.

**Figure 7:**
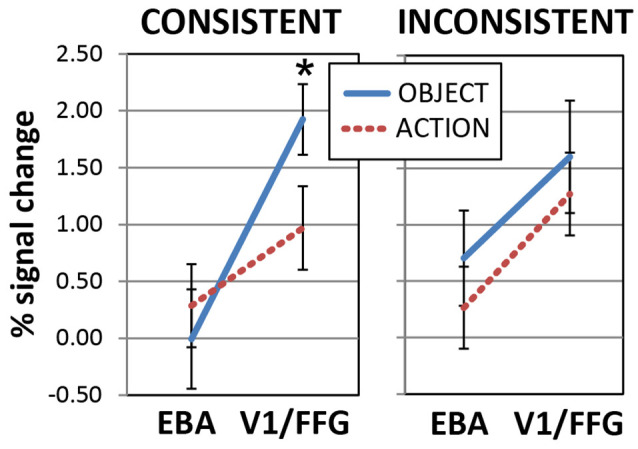
Responses to newly learnt action- and object-related spoken words in the primary visual cortex and fusiform gyrus (V1/FFG) and the extrastriate body area (EBA). Activations induced by words with a consistent **(Left)** or inconsistent **(Right)** meaning are plotted as a function of ROI. Note the larger responses to the newly learnt object than action word sounds in the V1/FFG area (Left), which is preferentially activated by object pictures. The opposite trend appears to emerge in EBA (which, by contrast, exhibited specific sensitivity to pictures of hand-related action pictures), although the *post hoc* comparison was not significant there. Responses to inconsistent-meaning items (Right) showed a main effect of ROI but no effects of semantic category (data from left-hemisphere’s ROIs labeled “*b*” and “*d*′’ in Figure 6. Error bars indicate SE). *p < 0.05.

Figure 6 shows a summary of the results. A repeated-measure ANOVA with factors Hemisphere, WordType, Consistency and ROI run on data from bilateral EBA and V1/FFG regions revealed a main effect of Hemisphere (*F*_(1,19)_ = 17.4, *p* = 0.0005) and a WordType-by-ROI interaction (*F*_(1,19)_ = 4.5, *p* = 0.048). As the left hemisphere showed the strongest signal (average % signal change in the two right-hemisphere ROIs overall did not differ from baseline: *F*_(1,19)_ = 0.50, *p* > 0.48, n.s., whereas those in the left-hemispheric ROIs did, *F*_(1,19)_ = 9.91, *p* < 0.01), we restricted the analysis to that hemisphere. An ANOVA run on the two ROIs “b” and “d” in the left hemisphere (data plotted in Figure 7) revealed an interaction of WordType, Consistency and ROI (*F*_(1,19)_ = 7.4, *p* = 0.013) and a main effect of ROI (*F*_(1,19)_ = 13.4, *p* = 0.002).

A separate ANOVA run on the consistent-only data set (left plot in Figure 7) confirmed the interaction of WordType-by-ROI (*F*_(1,19)_ = 8.0, *p* = 0.011) and the main effect of ROI (*F*_(1,19)_ = 14.5, *p* = 0.001). Planned comparisons confirmed the larger responses to newly-learnt (consistent) object-than to action-related spoken words in the left V1/FFG area (*t*_(19)_ = 2.2, *p* = 0.019, one-tailed, FWER corrected, α = 0.025), while EBA activations did not differ between the two semantic categories (*t*_(19)_ = 0.76, *p* > 0.45, n.s.). A similar ANOVA run on the inconsistent-meaning data (Figure 7, right plot) revealed no interaction and confirmed a main effect of ROI (*F*_(1,19)_ = 8.8, *p* = 0.008).

## Discussion

Auditory presentation of newly learnt spoken words activated left-lateralized superior temporal cortex and, after they had co-occurred with different exemplars from the same conceptual category (for example, four different cats), the novel sounds also sparked visual cortex, including left posterior fusiform and bilateral *primary* visual cortex (BA 17). Such visual cortex activation was specific to novel word forms associated with a basic semantic category (objects), as hearing these spoken items elicited significantly stronger visual responses than novel words previously paired with specific types of action. Intriguingly, words associated with a wide range of objects (or actions) did not significantly activate the occipital regions, either. These results document the formation of associative semantic links between a novel spoken word form and a basic conceptual category (i.e., that of a familiar animal), localizing, for the first time, brain correlates of the newly acquired word meaning to the primary visual cortex.

At the semantic level, our experiment modeled features of early stages of language learning, where words are semantically grounded in objects and actions. More precisely, the word form novel to the infant is being used by the adult in temporal vicinity to referent objects. Brain-constrained neural-network simulations indicate that the correlated activity in visual and linguistic areas brought about by such scenarios leads to synaptic strengthening between neurons in widespread areas of the network (Garagnani and Pulvermüller, 2016; Tomasello et al., 2017, 2018). As such modeling results demonstrate, the distributed word circuits built by linguistic-perceptual correlations should span perisylvian language areas in inferior-frontal and superior-temporal cortex along with the ventral visual stream, reaching into early—including primary—visual cortex. Our present results fully confirm the model’s predictions insofar as such early visual areas are concerned. In particular, contrary to diverging results from studies of the processing of first languages acquired early in life (see “Introduction” section), the present learning experiment shows that the repeated co-perception of novel spoken word forms and visual objects of one semantic type changes neuronal connectivity in such a way that, after learning, the word sounds selectively reactivate primary visual cortex (V1). This visual activation goes hand-in-hand with the fact that the word forms have specific visually-related “meaning.”

Our study falls short of addressing several relevant aspects of semantics. For example, knowledge about meaning is acquired also when the learner hears (or reads) multiple word forms in texts and conversations: using correlated neuronal activity, this leads to combinatorial, distributional information being stored in the brain, which contributes to semantic knowledge. Although looking in detail at word-object relationships relevant in the context of semantic grounding, the present work did not attempt to tackle this aspect.

Any pre-established links between word forms and “content” in the widest sense were ruled out by meticulous counterbalancing of all word forms used across learning conditions and subjects (see “Materials and Methods” and Supplementary Material). This was done, in particular, to remove possible influences of phonological shape on semantic processing, as it might be due to physically-motivated semantic features (such as that lower pitch may index bigger things), possibly genetically co-determined sound symbolism (e.g., the pseudoword “maluma” being perceived as matching a round but not an edgy shape) or language-specific phonotactic preferences (Dingemanse et al., 2015). These and many others in a wider-sense semantic properties certainly play a role in language processing but were not considered here.

One important feature that the current study did attempt to address is action semantics. Wittgenstein’s claim that language is woven into action and thereby receives part of its meaning was modeled in our elementary learning experiment by co-presenting novel spoken words with pictures of actions. These were either from one specific action type characterized by movement features, aim and action-related objects—for example grasping (different objects) or pouring—or from the wider set of human object-related body actions. In both cases (learning of “basic action categories” and meanings of wider action spectrum type) our behavioral results indicated lower success in learning word-action picture contingencies. The reduced ability of participants to recognize novel words with action- than object-related meaning (see Figure 2) may relate to a range of different reasons, which we speculate may include the following: (1) to avoid distracting our subjects from the important action features depicted, we tried to keep the action pictures of one basic category very similar and took the photographs in the same environment and lighting. This led to lack of variability across action pictures, which may have made these stimuli less interesting and attention-capturing when compared with the colorful and variable animal pictures; (2) whereas animal pictures included one object on a background, typical action photographs had to include (part of) an actor (i.e., the hand/arm), a tool (hammer) and sometimes even a target object (nail). This made the action necessarily more complex than the object pictures. Furthermore, while images depicting animals are most straightforward to be classified into basic conceptual categories (particularly for mammals, which dominated our image sample), many of the action pictures may be classified as belonging to a range of plausible categories, at different levels of abstraction. For example, a “finger button-press” image (see samples in Supplementary Material) could be interpreted as a doorbell-ringing action, switching on/off a generic device (e.g., a light, a tape recorder, etc.), or even—if other buttons are visible—as choosing a set of possible alternatives. This made the task of identifying a suitable set of conceptual categories more challenging for the action pictures group, likely making the linguistic learning task harder (recall that participants were not explicitly told about the type of training they were being exposed to, or what the underlying conceptual categories were); and (3) language learning children seem to frequently adopt a strategy for relating novel word forms to whole objects (Bloom and Markson, 1998); if our participants adopted this strategy, a further possible reason for their difficulty in learning action meanings becomes apparent (see point 2. above). In essence, there are a range of plausible reasons that may have contributed to the less successful outcome of action words training. Nonetheless, participants’ discrimination index for this category—albeit lower than that for object-related words—was well above chance level (see Figure 2), indicating that participants were generally able to recognize action-related words, too. Intriguingly, the extrastriate body area (or EBA) strongly activated in our localizer task in response to the action pictures (see Figure 5A), suggesting that these images sparked brain processes related to body-part perception and possibly action. The trend towards relatively stronger activation in our EBA ROI to action words as compared with object words can only be taken as a “hint” of focal semantically-related brain processes unique to the former; still, the significant interaction due to stronger activation to a novel basic-category object than to action word sounds in early visual areas (and the opposite trend emerging in the EBA) provides strong support for focal activation signatures for the learnt animal word conceptual categories.

A range of predictions emerging from the results of our previous neurobiologically constrained simulations of semantic processing was not addressed here. So-called semantic hubs are supposed to activate in semantic processing regardless of which type of meaning features are being processed (Patterson et al., 2007). These areas, postulated, by different authors, in anterior- and posterior-temporal, inferior-parietal and inferior-frontal cortex (Pulvermüller, 2013), could have become active in the general contrast “trained vs. untrained” novel words. However, here this contrast did not yield reliable activation differences, possibly because not all words were successfully learnt (i.e., linked with an object or action information). Previous studies using words from languages acquired in early life showed category-specific activity differences in the posterior temporal cortex (Martin, 2007; Pulvermüller, 2013). Most notably, a series of studies reported specific activity in posterior-inferior temporal cortex to animal words (as compared with tool words; Chao et al., 1999; Martin, 2007). This activity was not prominent in the present dataset, although, as close inspection of Figure 5D reveals, significant left inferior-temporal activation was seen in the Consistent-Object words vs. Silence Contrast (MNI coordinates of peak voxel: *x* = −28, *y* = −60, *z* = −24, *T* = 6.4, *K_E_* = 1,530). Indeed, this activation cluster partly overlaps with the one produced in the left fusiform gyrus by the localizer task in response to the object pictures (see Table 2; only the margins are visible in Figure 5C).

The prominent feature of the present results is the striking activation of early (especially primary) visual cortices to newly learnt word sounds from the consistent-object semantic category. This activation is reminiscent of that reported by a pioneering study (Martin et al., 1996) in which right hemispheric activation in animal naming had been observed using positron emission tomography. The present work suggests that these early results, although to our knowledge not replicated by other studies using natural language stimuli, receive confirmation if all hardly controllable factors that might influence the processing of real-language words are excluded by stringent experimental design.

The fact that early and even primary sensory cortices can kick-in when processing aspects of semantics is of utmost importance for the current debate in cognitive neuroscience addressing the role of semantic grounding. As Harnad pointed out, the learning of the meaning of linguistic signs necessitates that at least a set of words are learnt in the context of objects and actions and that the connections are made between these symbols and what they are normally used to speak about (Harnad, 1990, 2012; Cangelosi et al., 2000). Symbolic conceptual theories sometimes try to ignore this fact and postulate a somewhat mysterious link between sign and concept, although it is generally agreed upon that, apart from basic sound-symbolic links, the pairings between word forms and the objects, actions and concepts they relate to, are entirely arbitrary. Thus, if a word relates to a concept, this relationship must have been established by learning. While various forms of learning (e.g., combinatorial, inferential, trial and error) might play a role, grounding the meaning of an initial set of words *via* the correlation between objects in the world and symbol occurrences is one important and necessary stage of language acquisition. We claim that there is no other way to provide semantic grounding of an initial, base vocabulary. Our current results show, for the first time, that it is indeed a link between language and meaning information in primary visual cortex that emerges as a result of the co-occurrence of words and objects in the world.

## Data Availability Statement

The raw data supporting the conclusions of this article will be made available by the authors, without undue reservation.

## Ethics Statement

The studies involving human participants were reviewed and approved by Ethics committee of the Charité University Hospital, Campus Benjamin Franklin, Berlin, Germany. The patients/participants provided their written informed consent to participate in this study.

## Author Contributions

MG and FP planned the experiment and wrote the main manuscript text. EK and MG conducted data collection and analysis. All authors contributed to the article and approved the submitted version.

## Conflict of Interest

The authors declare that the research was conducted in the absence of any commercial or financial relationships that could be construed as a potential conflict of interest.

## Footnotes

^1^https://surfer.nmr.mgh.harvard.edu/optseq/

^2^http://www.fil.ion.ucl.ac.uk/spm/
