# Supplementary material for: Semantic Grounding of Novel Spoken Words in the Primary Visual Cortex
Source: Front Hum Neurosci. 2021 Feb 24;15:581847. doi: 10.3389/fnhum.2021.581847 (PMC7959837; doi:10.3389/fnhum.2021.581847)
Supplement: Supplementary file 1 [file Data_Sheet_1.pdf]

Supplementary Material

S1 – Pseudoword Stimuli

We built eight sets (A, B, ... H) of 16 bi-syllabic pseudowords by combining one of six possible CC ([tʃ], [ʃR], [ts], [fl], [pf], [ʃl]) sequences and one of six possible stop-consonants amongst [b], [p], [d], [t], [g], [k] with two – possibly identical – vowels amongst a, e, o, u, i, ä, ü, ö. Specifically, the 16 CCV syllables S_1={tsha, shra, tse, fle, pfo, shlo, tshu, shru, tsi, fli, pfä, shlä, tshü, shrü, pfö, shlö} and 16 CV syllables S_2= {ba, pe, dü, to, gi, kö, bö, pu, de, tä, gu, ka, bä, pü, bo, pi}, all phonotactically legal in German, were used. Digital recordings (sampling rate 44.1 kHz) of 12 samples of each syllable in $S_1 \cup S_2$ spoken in random order by a female native German speaker were acquired in a soundproof room. From these, items having approximately the same F0 frequency were selected (average ± standard deviation, or SD: 224.3 ± 2.8 Hz for S_1; 225 ± 2.2 Hz for S_2) and normalized to the same maximum amplitude value (-17 dB for S_1, -19dB for S_2 items). Within each syllable set, the recordings were then carefully aligned to their respective "perceptual centres" (Morton, Marcus, & Frankish, 1976) – here, the vowel onset – and padded with silence, so as to obtain stimuli of equal duration (450ms for S_1, 290ms for S_2).

The final 128 pseudowords (see Supplementary Table 1 below) were made up by one of the 16 possible CCV syllables from S_1 followed by one of the 16 CV syllables from S_2, each syllable in $S_1 \cup S_2$ being used exactly once in each of the eight sets. To avoid differential coarticulation cues and minimize acoustic differences, the same second-position syllable recordings were cross-spliced the onto the same first-position ones, leaving a 100ms gap of silence between them (this produced 840ms-long stimuli). For the analysis and generation of the stimuli we used the open-source software Audacity (http://sourceforge.net/projects/audacity/).

Set A	Set B	Set C	Set D	Set E	Set F	Set G	Set H
Tshude	Tsede	Pfädü	Tshüdü	Tshudü	Tsedü	Pfäde	Tshüde
Shrudü	Fledü	Shläde	Shrüde	Shrude	Flede	Shlädü	Shrüdü
Pfäpi	Tshüpi	Tsepu	Tshupu	Pfäpu	Tshüpu	Tsepi	Tshupi
Shläpu	Shrüpu	Flepi	Shrupi	Shläpi	Shrüpi	Flepu	Shrupu
Tsetä	Tshutä	Tshüto	Pfäto	Tseto	Tshuto	Tshütä	Pfätä
Fleto	Shruto	Shrütä	Shlätä	Fletä	Shrutä	Shrüto	Shläto
Tshüba	Pfäba	Tshubö	Tsebö	Tshübö	Pfäbö	Tshuba	Tseba
Shrübö	Shläbö	Shruba	Fleba	Shrüba	Shläba	Shrubö	Flebö
Pfobo	Tshabo	Tsibä	Pföbä	Pfobä	Tshabä	Tsibo	Pföbo
Shlobä	Shrabä	Flibo	Shlöbo	Shlobo	Shrabo	Flibä	Shlöbä
Tsika	Pföka	Tshakö	Pfokö	Tsikö	Pfökö	Tshaka	Pfoka
Flikö	Shlökö	Shraka	Shloka	Flika	Shlöka	Shrakö	Shlokö
Tshagu	Pfogu	Pfögi	Tsigi	Tshagi	Pfogi	Pfögu	Tsigu
Shragi	Shlogi	Shlögu	Fligu	Shragu	Shlogu	Shlögi	Fligi
Pföpe	Tsipe	Pfopü	Tshapü	Pföpü	Tsipü	Pfope	Tshape
Shlöpü	Flipü	Shlope	Shrape	Shlöpe	Flipe	Shlopü	Shrapü

Supplementary Table 1. The complete set of auditory stimuli used. Each pair of pseudoword items in set A (or B, C, D) had a corresponding pair in set E (or F, G, H, respectively), which was identical except that the two second-position (CV) syllables were "switched" around. Note that these sets were defined in such a way that *any two items* in {A ∪ B ∪ C ∪ D} or in {E ∪ F ∪ G ∪ H} differed by at least 2 letters. Thus, the four Sets A,.., D (four left-hand side columns of) were always assigned either

all to the Trained or all to the Untrained condition, guaranteeing a between-item distance of at least 2 letters/phonemes in the set of stimuli to be learnt by any participant and, at the same time, ensuring a very strict matching.

S2 – Objects and Actions visual stimuli

Sixteen familiar objects (animals: bear, beetle, cat, cow, deer, dog, dove, fish, fly, fox, goat, horse, lion, mouse, pig, rabbit) and sixteen familiar hand actions (clap, draw, grasp, hammer, open, peel, pour, press, push, rip, scratch, saw, sew, stamp, stir, type) were selected. For each chosen animal type, four different pictures (depicting four different breeds) were used; similarly, for each hand action we used four different instances of the same gesture (e.g., grasping of different objects, viewed from different angles and performed by different actors). Whenever the action was performed single-handedly (e.g., drawing), we ensured that the picture portrayed a right-handed execution. All 64 hand action pictures were taken on site using a Sony Alpha 57 digital camera. All images were edited using the Adobe Photoshop software package (Adobe Systems Inc., San Jose, CA, USA) to improve clarity and remove non-relevant details. The pictures used for the experiment were matched on the following variables: familiarity of the subject depicted (on a scale from 1 to 7, with 1 = "Unfamiliar" and 7 = "Very familiar"; object: 5.40 ± 0.98; action: 4.97 ± 1.59; $F(1,11)=2.66$, $p>.13$), picture clarity (on a scale between 1 and 7, with 1 = "Overall unclear" and 7 = "Very clear"; object: 6.53 ± 0.46; action: 6.39 ± 0.45; $F(1,11)=.87$, $p>.37$), and "naming coherence" (computed as the % of participants providing the same – most frequently chosen – label as first descriptor for a picture; object: 93.1% ± 10.2%; action: 92.8% ± 7.7%; $t_{63}=0.16$, $p>.87$); ratings were provided by a group of 12 volunteers in a preliminary behavioural pilot. Furthermore, the (German) nouns and verb forms used most frequently to describe the 16 objects and 16 actions were matched for lemma frequency ($t_{15}=.56$, $p > .5$) (dlexDB, http://www.dlexdb.de/). We also produced "blurred" versions of all 128 images so as to make the subject of the original pictures unrecognisable while preserving colour information.

References

Morton, J., Marcus, S., & Frankish, C. (1976). Perceptual centers (P-centers). *Psychological Review, 83*(5), 405-408.

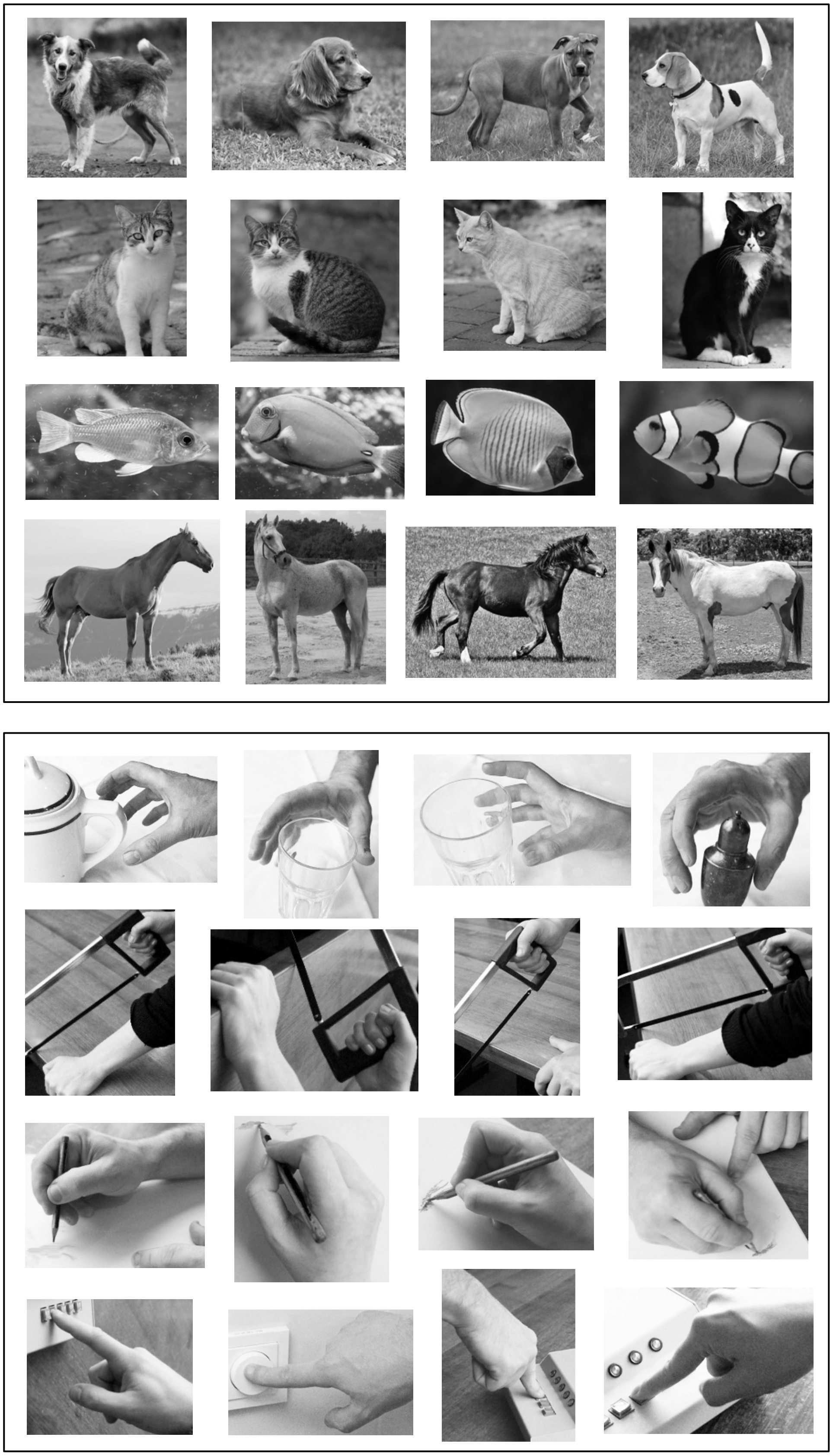

Supplementary Figure 1. Representative examples of object (top) and action (bottom) stimulus pictures used in the study.
